# Supplementary material for: Model building of protein complexes from intermediate-resolution cryo-EM maps with deep learning-guided automatic assembly
Source: Nat Commun. 2022 Jul 13;13:4066. doi: 10.1038/s41467-022-31748-9 (PMC9279371; doi:10.1038/s41467-022-31748-9)
Supplement: Supplementary file 1 — Supplementary Information [file 41467_2022_31748_MOESM1_ESM.pdf]

Supplementary Fig. 1

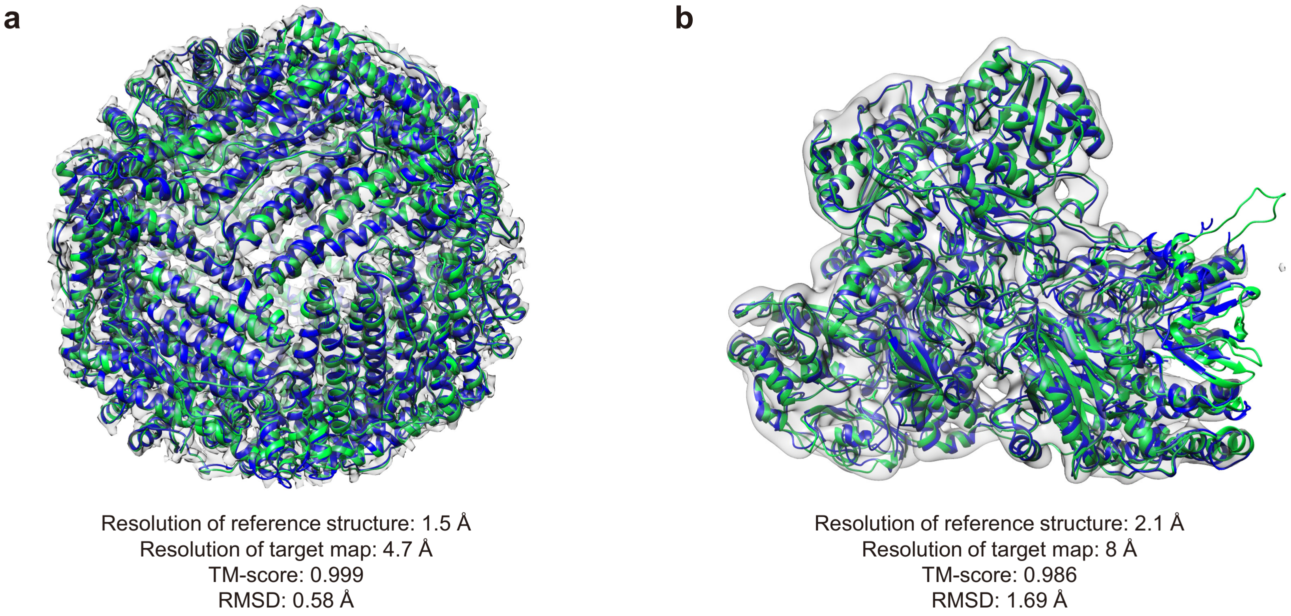

Supplementary Fig. 1: Evaluations of the EMBuild models built on lower resolution maps with respect to higher resolution reference structures. The reference PDB structures are colored in blue, the built structures by EMBuild are colored in green, and the corresponding EM density maps are colored in transparent gray. **a** EMD-2788 at 4.7 Å resolution. The reference structure is the X-ray diffraction structure at 1.5 Å resolution (2W0O). **b** Low-pass filtered map at 8.0 Å resolution from the half-maps of EMD-12661. The reference structure is the PDB structure associated with the 2.1 Å primary map of EMD-12661.

Supplementary Fig. 2

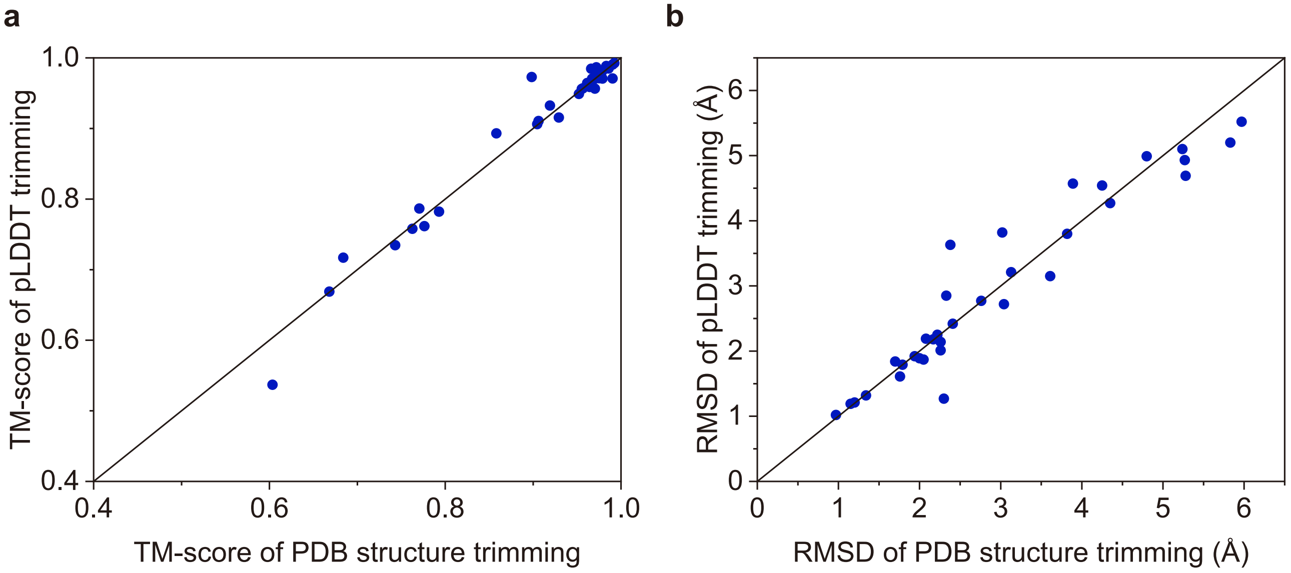

Supplementary Fig. 2: Comparison of the EMBuild models using PDB structure trimming and pLDDT trimming, on the test set of 34 single-particle EM maps. **a** TM-score. **b** RMSD. Source data are provided in the Source Data file.

Supplementary Fig. 3

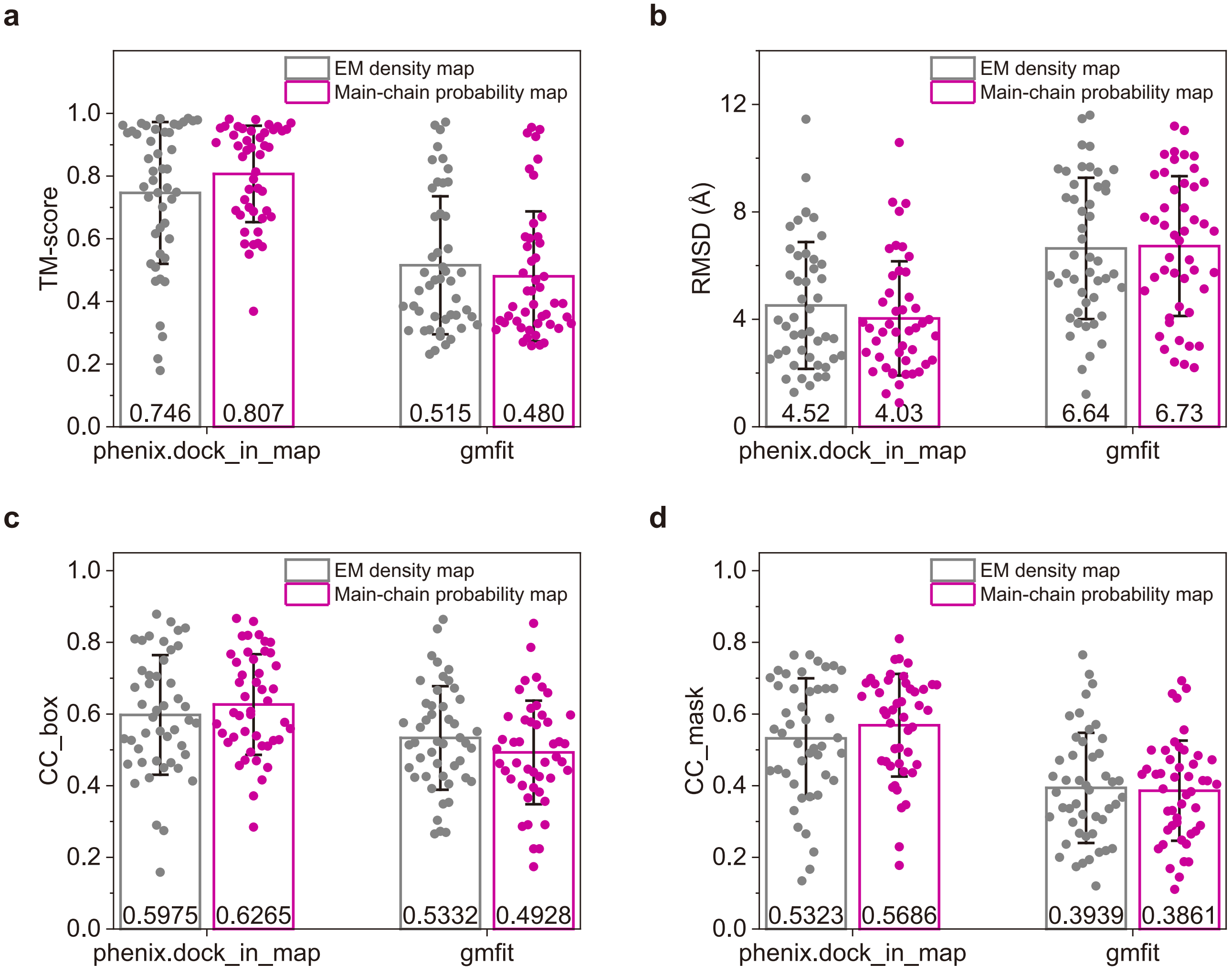

Supplementary Fig. 3: Comparison between the built models from the EM density maps and the main-chain probability maps for *phenix.map_to_model* and gmfit on the test set of 47 single-particle EM maps. **a–d** Average TM-score (**a**), RMSD (**b**), CC_box (**c**), and CC_mask (**d**) values among $n = 47$ individual test cases. The error bars indicate $+/-$ 1.0 standard deviations. Source data are provided in the Source Data file.

Supplementary Fig. 4

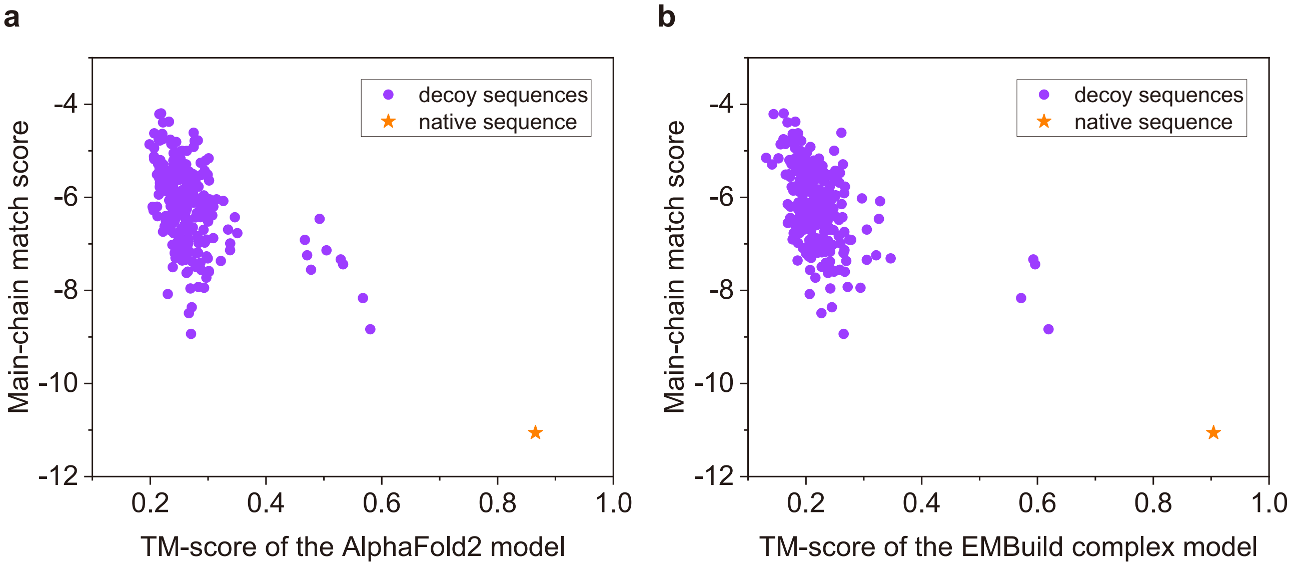

Supplementary Fig. 4: Evaluation of EMBuild in finding the native sequence from a pool of decoy sequences. The AlphaFold2 structures for 298 decoy sequences plus one native sequence are fitted into EMD-0290 by EMBuild to build a trimer model. The average main-chain match score of the three fitted chains is calculated for each sequence. **a** The relationship between the main-chain match scores and the TM-scores for the AlphaFold2-predicted structures of different sequences. **b** The relationship between the main-chain match scores and the TM-scores for the EMBuild-built trimer models of different sequences. Source data are provided in the Source Data file.

Supplementary Fig. 5

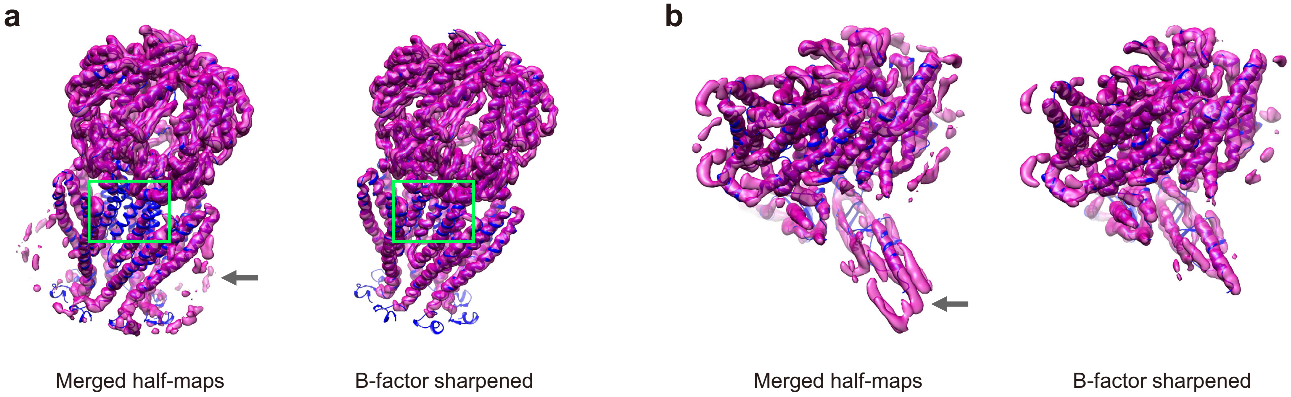

Supplementary Fig. 5: Comparison of the main-chain probability maps predicted from unsharpened merged half-maps and from B-factor sharpened map. Arrows indicate noises that are present in the main-chain probability map predicted from unsharpened half-maps. **a** EMD-20510. Highlighted by green boxes are the probability volumes that are significantly improved after applying B-factor sharpening. **b** EMD-4646.

Supplementary Fig. 6

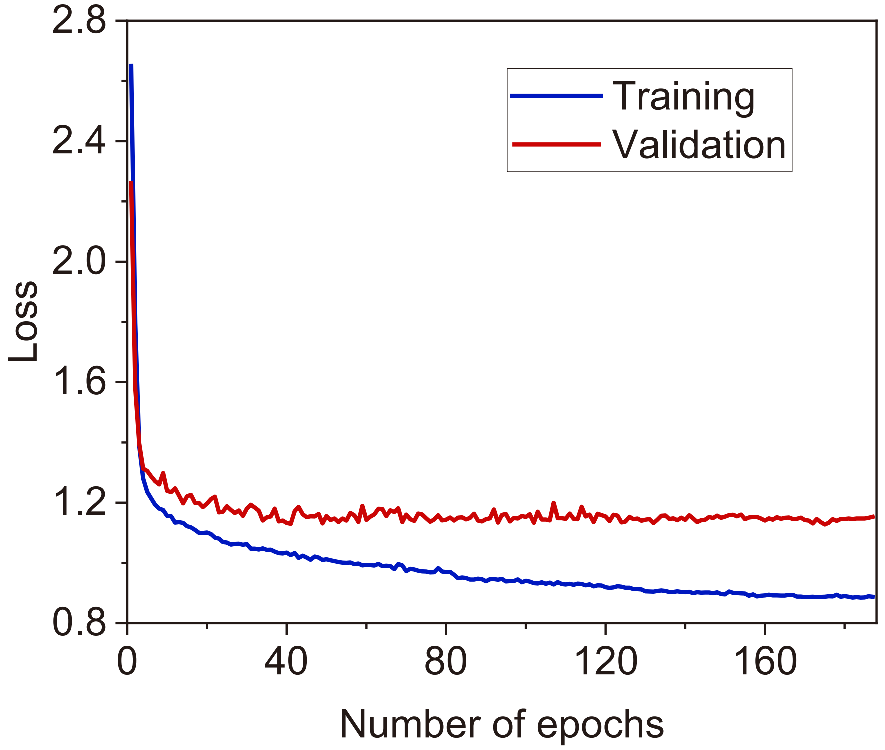

Supplementary Fig. 6: Learning curve of the UNet++ in main-chain probability prediction. The loss used here was the the sum of Smooth L1 loss and SSIM loss. Source data are provided in the Source Data file.
