# Supplementary material for: Model building of protein complexes from intermediate-resolution cryo-EM maps with deep learning-guided automatic assembly
Source: Nat Commun. 2022 Jul 13;13:4066. doi: 10.1038/s41467-022-31748-9 (PMC9279371; doi:10.1038/s41467-022-31748-9)
Supplement: Supplementary file 3 — Description of Additional Supplementary Files [file 41467_2022_31748_MOESM3_ESM.pdf]

Description of Additional Supplementary Files

File Name: Supplementary Data 1
Description: List of cases used to train the UNet++ in main-chain probability prediction.

File Name: Supplementary Data 2
Description: Evaluation results of the models built by different methods including EMBuild, phenix.dock_in_map, phenix.dock_in_map+main-chain probability map, DEMO-EM, DEMO-EM+AlphaFold2 structure, gmfit, and gmfit+main-chain probability map, on the test set of 47 single-particle cryo-EM maps.   "-" means no output for this test case, and "N/A" in FSC05 means that the case failed to give a valid FSC05 value.

File Name: Supplementary Data 3
Description: Evaluation results of the models built by different methods including EMBuild, phenix.dock_in_map, and gmfit, on the test set of 16 tomogram averaging cryo-EM maps. "-" means no output for this test case, and "N/A" in FSC05 means that the test case failed to give a valid FSC05 value.

File Name: Supplementary Data 4
Description:

a. Coordinates-only quality check results of EMBuild models on the test set of 47 single-particle EM maps.
b. Coordinates-only quality check results of EMBuild models on the test set of 16 subtomogram average EM maps.

File Name: Supplementary Data 5
Description: Comparison of the EMBuild models with and without using symmetry information, on the test set of 19 single-particle EM maps with C or D symmetry.

File Name: Supplementary Data 6
Description: Comparison of the EMBuild models using PDB structure trimming and pLDDT trimming, on the test set of 34 single-particle EM maps.

File Name: Supplementary Data 7
Description: Evaluation results of using EMBuild to find the native sequence from a pool of 298 decoys sequences plus one native sequence.

File Name: Supplementary Data 8
Description: Running times of different methods. The running times for main-chain probability map predictions are measured on four NVIDIA A100 GPUs. Different methods are compared on Intel(R) Xeon(R) Gold 6240. Main-chain probability map prediction is the prerequisive for EMBuild, and gmconvert is the prerequisive for gmfit. EMBuild is evaluated both on a single thread and on 36 threads in parallel using OpenMP. The other methods are evaluated with a single thread.
