# Supplementary material for: Model building of protein complexes from intermediate-resolution cryo-EM maps with deep learning-guided automatic assembly
Source: Nat Commun. 2022 Jul 13;13:4066. doi: 10.1038/s41467-022-31748-9 (PMC9279371; doi:10.1038/s41467-022-31748-9)
Supplement: Supplementary file 12 — Reporting Summary [file 41467_2022_31748_MOESM12_ESM.pdf]

nature portfolio

Corresponding author(s): Sheng-You Huang

Last updated by author(s): Jun 16, 2022

Reporting Summary

Nature Portfolio wishes to improve the reproducibility of the work that we publish. This form provides structure for consistency and transparency in reporting. For further information on Nature Portfolio policies, see our Editorial Policies and the Editorial Policy Checklist.

Statistics

For all statistical analyses, confirm that the following items are present in the figure legend, table legend, main text, or Methods section.

n/a	Confirmed	
☐	☒	The exact sample size (*n*) for each experimental group/condition, given as a discrete number and unit of measurement
☒	☐	A statement on whether measurements were taken from distinct samples or whether the same sample was measured repeatedly
☒	☐	The statistical test(s) used AND whether they are one- or two-sided<br>*Only common tests should be described solely by name; describe more complex techniques in the Methods section.*
☒	☐	A description of all covariates tested
☒	☐	A description of any assumptions or corrections, such as tests of normality and adjustment for multiple comparisons
☐	☒	A full description of the statistical parameters including central tendency (e.g. means) or other basic estimates (e.g. regression coefficient) AND variation (e.g. standard deviation) or associated estimates of uncertainty (e.g. confidence intervals)
☒	☐	For null hypothesis testing, the test statistic (e.g. *F*, *t*, *r*) with confidence intervals, effect sizes, degrees of freedom and *P* value noted<br>*Give P values as exact values whenever suitable.*
☒	☐	For Bayesian analysis, information on the choice of priors and Markov chain Monte Carlo settings
☒	☐	For hierarchical and complex designs, identification of the appropriate level for tests and full reporting of outcomes
☒	☐	Estimates of effect sizes (e.g. Cohen's *d*, Pearson's *r*), indicating how they were calculated

Our web collection on statistics for biologists contains articles on many of the points above.

Software and code

Policy information about availability of computer code

Data collection	Greedy algorithm for dataset clustering (written in-house).
Data analysis	AlphaFold (https://github.com/deepmind/alphafold/);<br>Chimera (version 1.15, https://www.cgl.ucsf.edu/chimera/) for data visualization;<br>DEMO-EM (https://zhanggroup.org/DEMO-EM/);<br>EMBuild (version 1.0, http://huanglab.phys.hust.edu.cn/EMBuild/) developed in this study.<br>gmfit (LastModified:2019/09/21, https://pdbj.org/gmfit/);<br>MM-align (version 20210816, https://zhanggroup.org/MM-align/);<br>MolProbity (used via Phenix, http://molprobity.biochem.duke.edu/);<br>MonoDir (used via Scipion v3.0.10, http://scipion.i2pc.es/);<br>Phenix (version 1.19.2-4158, https://phenix-online.org/);<br>Python (version 3.8.8, https://www.python.org/);<br>PyTorch (version 1.8.1+cu111, https://pytorch.org/);<br>RELION (version 3.1.3, https://github.com/3dem/relion);<br>STRIDE (http://webclu.bio.wzw.tum.de/stride/);<br>SWORD (version 1.0, https://www.dsimb.inserm.fr/sword/).

For manuscripts utilizing custom algorithms or software that are central to the research but not yet described in published literature, software must be made available to editors and reviewers. We strongly encourage code deposition in a community repository (e.g. GitHub). See the Nature Portfolio guidelines for submitting code & software for further information.

Data

Policy information about availability of data

All manuscripts must include a data availability statement. This statement should provide the following information, where applicable:

- Accession codes, unique identifiers, or web links for publicly available datasets
- A description of any restrictions on data availability
- For clinical datasets or third party data, please ensure that the statement adheres to our policy

Source data are provided with the Article, Supplementary information, Supplementary Data, and Source Data File. Source Data File are provided with this paper. All published data sets used in this paper were taken from the EMDB and PDB (accession codes specified in the figure captions and the tables in Supplementary Data). Other data that support the findings of this study are available from the corresponding author upon request.

Field-specific reporting

Please select the one below that is the best fit for your research. If you are not sure, read the appropriate sections before making your selection.

[x] Life sciences [ ] Behavioural & social sciences [ ] Ecological, evolutionary & environmental sciences

For a reference copy of the document with all sections, see nature.com/documents/nr-reporting-summary-flat.pdf

Life sciences study design

All studies must disclose on these points even when the disclosure is negative.

Sample size	We have collected a non-redundant dataset of cryo-EM maps from the EMDB. All the single-particle EM entries at 4–8 Å resolution that have associated PDB models were downloaded from EMDB (https://www.ebi.ac.uk/emdb/) and PDB(http://www.pdbj.org/). The EM map and its corresponding PDB structure that have any of the following features were removed: (i) without side chain atoms, (ii) including unknown residues (UNK), (iii) including missing chain or non-protein chain, (iv) having nonorthogonal map axis, (v) corresponding to multiple PDB or EMDB entries, and (vi) having severe misfits between the PDB model and EM map. In order to ensure the feature (vi), we calculated the cross-correlation between the deposited map and the map simulated from the PDB model at the same resolution using the UCSF chimera. Any map and its associated PDB model that have a cross-correlation of less than 0.65 were excluded. Afterwards, all the remaining maps were manually checked. The remaining cases were retained as the initial dataset. To remove redundancy, the initial dataset of cases was clustered using greedy algorithm. Two models are considered to be similar if any chain in the first model has > 30% sequence identity with any chain in the second model. The one with the largest number of similar cases is chosen as the representative of the corresponding cluster, and then the rest cases in the cluster are removed. This procedure is repeated until all the cases are clustered. The resulted non-redundant training set consists of the representatives of each cluster. A total of 262 pairs of EM maps and associated PDB structures were retained. In order to train a deep learning model for predicting main-chain probability map, 209 maps were randomly selected as the final training set (Supplementary Data 1).<br>To build an independent test set with a sufficient number of valuable cases, all the cases in the initial dataset that have > 30% sequence identity with any case in the training set are excluded. Then, any pair of EM map and its associated PDB structure that meet the following criteria are removed: (i) having only one chain and (ii) having more than 30% gap in the structure according to the gene sequence. For saving time in evaluations, we have also removed those cases with more than 10 chains, though our method can work with any number of chains. The remaining cases are clustered using a sequence identity cutoff of 70% by the similar greedy algorithm described above. The final test set contains 47 single-particle cryo-EM maps with resolutions ranging from 4.0 to 8.0 Å (Supplementary Data 2).<br>To evaluate the performance of EMBuild on the maps by subtomogram averaging of cryo-ET data, we further constructed another independent test set of subtomogram averaging maps. All the subtomogram averaging maps with resolutions within 10.0 Å that have associated PDB models are filtered and clustered using the same way as that for the test set of single-particle maps, except for allowing more than 10 chains. The final test set contains 16 subtomogram averaging maps with resolutions ranging from 3.7 to 9.3 Å (Supplementary Data 3).
Data exclusions	When applying AlphaFold2, the corresponding PDB structure of the given sequence was excluded from the templates by setting the "max_template_data" to the day before the released date of the corresponding PDB structure.
Replication	All results could be reproduced by the downloadable package of EMBuild, or based on the Methods/Supporting Information.
Randomization	The training set and test set of cryo-EM maps and their associated deposited PDB structures were randomly collected from the EMDB and PDB, after the consideration of non-redundancy within each set and between training and test sets.
Blinding	Blinding is not necessary in this study because the experiments are based on preexisting data from other sources, the analysis is down by software without human intervention and the results are not expected to be influenced by subjective factors.

Reporting for specific materials, systems and methods

We require information from authors about some types of materials, experimental systems and methods used in many studies. Here, indicate whether each material, system or method listed is relevant to your study. If you are not sure if a list item applies to your research, read the appropriate section before selecting a response.

Materials & experimental systems

n/a	Involved in the study
☒	☐ Antibodies
☒	☐ Eukaryotic cell lines
☒	☐ Palaeontology and archaeology
☒	☐ Animals and other organisms
☒	☐ Human research participants
☒	☐ Clinical data
☒	☐ Dual use research of concern

Methods

n/a	Involved in the study
☒	☐ ChIP-seq
☒	☐ Flow cytometry
☒	☐ MRI-based neuroimaging
